# Galacto-oligosaccharide production by *Lactobacillus delbrueckii* ssp. *bulgaricus* whole cells and lysates

**DOI:** 10.3168/jdsc.2024-0580

**Published:** 2024-06-13

**Authors:** Giselle K.P. Guron, Arland T. Hotchkiss, John A. Renye, Adam M. Oest, Michael J. McAnulty

**Affiliations:** Dairy and Functional Foods Research Unit, Eastern Regional Research Center, Agricultural Research Services, US Department of Agriculture, Wyndmoor, PA 19038

## Abstract

β-Galactosidase is currently applied in foods for reduction of lactose but can also be used for its transgalactosylation activity to synthesize galacto-oligosaccharides (GOS) as prebiotics. The ability of GRAS-status *Lactobacillus delbrueckii* strains to exhibit such activities would benefit consumers given their extensive history with dairy products. The objective of this study was to characterize the production of GOS in 6 *L. delbrueckii* strains for their ability to synthesize GOS in 50 m*M* sodium phosphate (pH 6.5) with a high lactose concentration of 788.8 m*M* at 50°C. *Lactobacillus delbrueckii* ssp. *bulgaricus* B548, LB11, and YB1 lysates released the most glucose at 112.8 ± 6.2 m*M*, 150.4 ± 11.7 m*M*, and 190.2 ± 12.2 m*M*, respectively. However, the ratio of free glucose to galactose released by B548 lysate (1.4 ± 0.3) was lower than that of LB11 (2.8 ± 0.6) and YB1 (2.5 ± 0.6) lysates, so the latter strains were screened at different initial lactose concentrations. The GOS yield from YB1 was not dependent on initial lactose concentration, averaging 54.3 ± 0.6% across starting lactose concentrations. However, optimal LB11 transgalatosylation had an initial concentration of 394.4 m*M* lactose instead of 788.8 m*M*, resulting in a GOS yield of 56.8% instead of 47.3%. In all cases for LB11, the lysates had greater free glucose, galactose, and GOS yield than whole cells. The ability of 2 *Lactobacillus delbrueckii* ssp. *bulgaricus* strains to produce GOS from common lactose-containing ingredients can have a range of applications in the dairy industry.

## Highlights

• *Lactobacillus bulgaricus* LB11 and YB1 produce GOS from 131.5 to 788.8 mM lactose at 50°C.
• LB11 and YB1 lysates produce greater GOS than whole cells.
• GOS yield by LB11 lysate was optimal at 394.4 mM lactose.
• Total GOS yield by YB1 lysate correlated with increased initial lactose.

## Graphical Abstract

**Figure:**
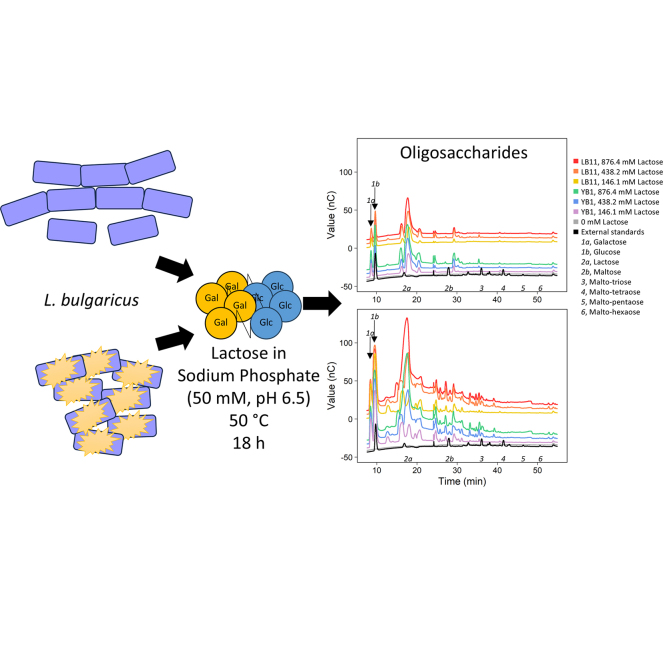
**Summary:** The whole cells and lysates of different strains of *Lactobacillus delbrueckii* ssp. bulgaricus were screened for the ability to synthesize galacto-oligosaccharides (GOS) through the transgalactosylation activities of their β-galactosidase (β-gal) at 50°C for 18 hours. The β-gal reactions from L. bulgaricus strains LB11 and YB1 were performed in increasing concentrations of lactose (146.1 to 876.4 mM) to determine which is optimal for the 2 different strains. The GOS were detected from whole cell and lysate reactions, although generally, the lysates yielded more GOS of varying degrees of polymerization.

The incorporation of oligosaccharides derived from milk carbohydrates into human diets provides a wide variety of prebiotic benefits for consumers, including promoting the growth of *Bifidobacterium* (Knol et al., 2005), which improves overall gastrointestinal health (Mei et al., 2022). Human milk oligosaccharides, which are present in breast milk, aid in the initial colonization of infant intestinal microbiomes (Masi and Stewart, 2022). Galacto-oligosaccharides (**GOS**), which can be synthesized from lactose derived from milk of any origin (Iqbal et al., 2022), can be added to infant formula, and may also be administered as a prebiotic to benefit hosts through adulthood (Mei et al., 2022). In addition to direct human health benefits, GOS can be synthesized from byproducts of dairy processes, such as sweet whey (Fischer and Kleinschmidt, 2021), thus upcycling products that would otherwise be wasted. Developing and optimizing strategies for GOS production could allow the dairy industry to satisfy market demands for health and wellness foods as well as provide more value for dairy byproducts.

Galacto-oligosaccharides consist of 2 or more galactose residues and are generally synthesized enzymatically using β-galactosidase (**β-gal**; Torres et al., 2010). β-Galactosidase is necessary to hydrolyze lactose into glucose and galactose, and it is capable of transgalactosylation when the acceptor is an additional sugar moiety instead of water (Torres et al., 2010). The extent to which these 2 reactions occur depends on the β-gal source, starting lactose concentration, pH, concentration of certain metal ions, and temperature (Torres et al., 2010; Guerrero et al., 2015). β-Galactosidase used for food ingredients typically comes from microbial sources, such as *Kluyveromyces lactis*, *Aspergillus oryzae*, or *Bacillus circulans*, each of which produce different profiles of GOS including different linkages and degrees of polymerization (Guerrero et al., 2015; Yin et al., 2017). These differences in GOS structure are likely to result in different prebiotic properties (van Leeuwen et al., 2016).

Other microbial species have been shown to produce a β-gal capable of producing GOS (Iqbal et al., 2022), with a good deal of interest in lactic acid bacteria (**LAB**) to produce GOS from milk-derived ingredients since they are considered generally regarded as safe (**GRAS**). In particular, Fara et al. (2020) found that 11 out of 20 *Lactobacillus delbrueckii* ssp. *bulgaricus* strains synthesized GOS after 24 h at 37°C with modified de Man, Rogosa, and Sharpe (**MRS**) containing 300 g/L lactose instead of glucose. Three out of 8 *Lactiplantibacillus plantarum* (previously known as *Lactobacillus plantarum*) strains also produced GOS under the same conditions (Fara et al., 2020). The β-gal from other *L. plantarum* strains were able to produce GOS at 50°C under neutral pH conditions (Iqbal et al., 2010; Gobinath and Prapulla, 2014). These GOS structures derived from *Lactobacillaceae* could potentially be specifically favored by the *Lactobacillaceae* themselves. For example, *L. bulgaricus* CRL450 had better growth with its own GOS as the sole carbon source compared with GOS produced by *Aspergillus oryzae*, which supports the need to identify additional GOS-producing strains more closely related to known probiotics species (Fara et al., 2020).

Activity of LAB β-gal varies by species and strain (Gomaa, 2018; Fara et al., 2020; Fischer and Kleinschmidt, 2021). Even among β-gal produced by *L. bulgaricus*, there are mutations among strains that can alter β-gal activity between LB43 and ATCC 11842 (Arsov et al., 2022). Screening for activity is often performed using colorimetric measurements of o-nitrophenyl-β-d-galactopyranoside (**ONPG**) hydrolysis or 5-bromo-4-chloro-3-indolyl-β-d-galactopyranoside (**X-gal**) cleavage (O'Hara and Kolida, 2016; Kolev et al., 2022). For GOS production, most processes use purified or overexpressed β-gal (Torres et al., 2010; Fischer and Kleinschmidt, 2018; Iqbal et al., 2022), but it was recently reported that GOS can be produced from whole cells of yogurt starter cultures using sweet whey or a sodium phosphate buffer containing lactose (Fischer and Kleinschmidt, 2021). It has also been previously reported that transgalactosylation can be favored in whole cells of *Bifidobacterium longum* over the lysate (Schwab et al., 2011). Whole cells could also potentially be useful in reducing mono- and disaccharides from crude GOS products (Pázmándi et al., 2021). The objective of this study was to characterize the production of GOS in 6 *L. delbrueckii* strains for their ability to synthesize GOS in 50 m*M* sodium phosphate (pH 6.5) containing high concentrations of lactose at 50°C.

Five *L. delbrueckii* ssp. *bulgaricus* strains and one *Lactobacillus delbrueckii* ssp. *lactis* strain (Table 1) were streaked onto MRS agar (Difco, Franklin Lakes, NJ) from frozen stock cultures (−70°C storage in 15% glycerol) and incubated at 37°C for 2 d. Individual colonies were inoculated in 5 mL of MRS broth and incubated at 37°C for 2 d. Cells were rinsed in an equal volume of 0.1% peptone (Difco) following centrifuged at 10,000 × *g* for 2 min at room temperature. Modified MRS with no dextrose (United States Biological, Salem, MA) supplemented with 10 g/L lactose (Spectrum Chemical MFG Corp., New Brunswick, NJ) (Fischer and Kleinschmidt, 2021) was filter sterilized through 0.2-µm polyethersulfone membranes (Thermo Fisher Scientific, Waltham, MA), and then used to resuspend rinsed cultures in an equal volume.

**Table 1:** Effects of cell preparation on β-galactosidase activity in 50 m*M* sodium phosphate (pH 6.5) with 788.8 m*M* lactose after 18 h at 50°C^1^

Strain	Glucose^2^ (m*M*)		Galactose^3^ (m*M*)		Glc:Gal^4^		GOS yield^5^ (%)	
	Whole cells	Lysate	Whole cells	Lysate	Whole cells	Lysate	Whole cells	Lysate
NRRL B548	22.0 ± 6.2efg	112.8 ± 2.7^c^	23.5 ± 7.9^d^	81.1 ± 13.3^a^	1.0 ± 0.2^cd^	1.4 ± 0.3bcd	38.3	39.4
NRRL B734	2.8 ± 0.1^h^	12.3 ± 0.3fgh	6.0 ± 0.5^d^	6.2 ± 0.3^d^	0.5 ± 0.0^d^	2.0 ± 0.1^ab^	31.8	34.5
LB11	30.8 ± 11.7^ef^	150.4 ± 11.1^b^	25.2 ± 8.1^cd^	56.0 ± 15.5^ab^	1.2 ± 0.2bcd	2.8 ± 0.6^a^	37.4	47.3
LB16	75.2 ± 1.5^d^	39.8 ± 2.2^e^	28.7 ± 4.5bcd	15.4 ± 2.0^d^	2.7 ± 0.3^a^	2.6 ± 0.3^a^	42.4	40.3
YB1	103.9 ± 6.2^c^	190.2 ± 12.3^a^	54.9 ± 10.0abc	78.7 ± 23.7^a^	1.9 ± 0.1abc	2.5 ± 0.6^a^	47.0	53.9
LDL20	1.0 ± 0.0^h^	3.8 ± 0.3^gh^	1.0 ± 0.4^d^	2.0 ± 0.2^d^	1.0 ± 0.3bcd	1.8 ± 0.3^ab^	42.2	37.0

a–h Different letters indicate significant differences among strains and cell preparations for each carbohydrate (*P* < 0.05, 2-way ANOVA, n = 3).

1 Glucose, galactose, and Glc:Gal values are mean ± SD (n = 3).

2 Measured using the Megazyme d-Glucose Assay Kit (GOPOD Format; Neogen).

3 Measured using the Megazyme Lactose and d-Galactose (Rapid) Assay Kit (Neogen).

4 Ratio of the mean glucose released to mean galactose released.

5 GOS yield (%) = [GOS peak heights/peak heights (galactose + glucose + lactose + GOS peaks)] × 100; GOS peaks were all peaks excluding galactose, glucose, and lactose.

To initially quantify β-gal activity, hydrolysis of ONPG was measured. The optical density (**OD**) at 675 nm (OD_675_) of overnight cultures was measured using the BioTek Cytation 5 (Agilent Technologies, Santa Clara, CA). One milliliter of each culture in triplicate was centrifuged at 10,000 × *g* for 2 min at room temperature, and the pellets were stored frozen at −70°C. The pellets were then resuspended in 1 mL of Z buffer (16.1 g/L Na_2_HPO_4_·7H_2_O, 5.5 g/L NaH_2_PO_4_·H_2_O, 0.75 g/L KCl, 0.25 g/L MgSO_4_·7H_2_O, 2.7 mL/L β-mercaptoethanol) containing 1% toluene. The resuspended pellets were incubated at room temperature for 10 min and incubated at 50°C for 10 min before adding 200 µL of ONPG (4 g/L Z buffer) solution. The solution was incubated for 4 min at 50°C, and then 300 µL of Na_2_CO_3_ was added to stop each reaction. The samples were centrifuged at 3,400 × *g* for 4 min at room temperature, and 200 µL of each supernatant was measured with the Cytation 5 (OD_420_). Miller units (U/mL) are defined as A_420_ × 1,000/4 min × OD_675_, where A_420_ is absorbance at 420 nm.

Strains were then prepared as either whole cell inoculums or as lysates for the transgalactosylation reactions. One hundred microliters was inoculated into 10 mL of modified MRS supplemented with 10 g/L lactose and incubated for 20 to 24 h at 37°C. The cultures were centrifuged at 10,000 × *g* for 5 min at 4°C. The pellets were rinsed with 3 mL of sodium phosphate, centrifuged, and resuspended in 1 mL of sodium phosphate and transferred to 2-mL Eppendorf tubes. One-milliliter aliquots for lysate preparation were stored at −70°C, whereas the whole cell preparations for GOS synthesis were used immediately. Initial aerobic plate counts ranged from 5.63 ± 0.01 cfu/mL to 8.47 ± 0.18 cfu/mL.

In a method modified from Haswell et al. (2013) and Sangwan et al. (2015) to prepare lysates, cells were thawed from freezer storage, and 0.1-mm zirconia beads (Invitrogen, Waltham, MA) were used to disrupt cells by vortexing on maximum for 1 min, followed by placement on ice for 30 s (repeated 9 more times). The debris was then separated by centrifugation (10,000 × *g*, 4°C, 10 min), and collected supernatants were further assessed for GOS production immediately.

To start the β-gal reactions in triplicate, 900 µL of 50 m*M* sodium phosphate (pH 6.5) supplemented with 876.4 m*M* lactose was added into 1.5-mL Eppendorf tubes, followed by 100 µL of whole cells or lysate preparations for all LAB strains for an initial lactose concentration of 788.8 m*M* for the 1-mL reaction. The reactions were incubated at 50°C for 18 h. Whole cell preparations were enumerated by plating 100 µL on duplicate MRS agar plates. The reactions were then heated for 5 min at 100°C to deactivate the enzymes. Debris was separated by centrifugation (10,000 × *g*, 4°C, 10 min). Free glucose and galactose were measured using the Megazyme d-Glucose Assay Kit (GOPOD format; Neogen, Lansing, MI) and Megazyme Lactose and d-Galactose (Rapid) Assay Kit (Neogen), respectively. Two-way ANOVA was performed using R (R Core Team, 2021) to determine significance (α < 0.05).

The GOS analysis was performed according to Yin et al. (2017). Samples were diluted 100-fold with deionized H_2_O before analysis by high-performance anion-exchange chromatography with pulsed amperometric detection (**HPAEC-PAD**) using a Dionex ICS-3000 system (Thermo Fisher Scientific) with the carbohydrate quad potential waveform for integrated amperometry and a AgCl reference electrode. A CarboPac PA-1 column (4 × 250 mm, Dionex) and CarboPac PA-1 guard column (4 × 50 mm, Dionex) was operated at 0.8 mL/min with a gradient of (A) 100 m*M* NaOH, (B) 600 m*M* CH_3_COONa in 100 m*M* NaOH, (C) 50 m*M* CH_3_COONa, and (D) deionized H_2_O (Yin et al., 2017) using the following gradient conditions: 0 min—10% A, 5% C, 85% D; 25 min—40% A, 50% C, 10% D; 55 min—75% A, 25% B; 60 min—100% B; 69 min—10% A, 5% C, 85% D; and 15 min hold under these conditions before the next injection. External standards for galactose, glucose, and lactose were also injected. Peaks not identified by the retention times of the external standards were identified as GOS peaks. The GOS yield (%) {[total GOS peak heights/peak heights (galactose + glucose + lactose + GOS peaks)] × 100} was calculated from the chromatograms to approximate GOS production.

The initial screen for β-gal activity in Z buffer showed *L. lactis* LDL20 had over 2-fold greater activity (944.4 ± 108.9 U/mL) when compared with the other strains based on ONPG hydrolysis. The next highest activities were observed for *L. bulgaricus* YB1 (443.0 ± 7.3 U/mL) and B734 (83.7 ± 20.5 U/mL). The β-gal activities for *L. bulgaricus* B548, LB11, and LB16 were all below 10 U/mL. In previous studies surveying the GOS production of *Lactobacillaceae*, differing levels of ONPG hydrolysis were also observed (Fara et al., 2020; Fischer and Kleinschmidt, 2021). Fara et al. (2020) reported varying degrees of β-gal activity from 4 strains of *L. bulgaricus*, with one being significantly higher than the other 3.

These strains were then tested for hydrolysis of lactose and transgalactosylation in a starting lactose concentration of 788.8 m*M* (270 g/L) in sodium phosphate at 50°C (Table 1). Generally, the lysates released more glucose into solution compared with their whole cell counterparts. This may be due to live organisms metabolizing the glucose hydrolyzed from lactose (Hickey et al., 1986). One exception was LB16 where 35.4 m*M* more glucose was released by the whole cells compared with their lysate (*P* < 0.001, 2-way ANOVA). The LB16 strain was also the only one that did not start with an initial cell concentration above 7 log_10_ cfu/mL, which could also contribute to differences in activity between whole cells and lysates compared with the other strains.

Aerobic plate counts on MRS agar were measured at the end of the 18-h incubation period to determine if the cells remained viable within the high lactose concentration at 50°C, but no growth was detected. However, since culturable cells were not enumerated between the initial and 18-h time points, it is unknown for how long cells remained intact. Alternatively, the β-gal of injured cells that are no longer culturable may still be active. Since β-gal activity is intracellular, it is not clear whether glucose and GOS are released into solution via a sugar transporter or leakage from injured or dead cells, although strains that produce a lactose permease may be able to transport some GOS structures in and out of the cell, depending on concentration gradients (Andersen et al., 2011).

Free galactose was also measured, but unlike with free glucose, there were not as many significant differences among the strains and cell preparations (Table 1). The galactose released by the *L. bulgaricus* B548 lysates was 57.6 m*M* greater than galactose released by the whole cells (*P* < 0.001, 2-way ANOVA), which was the greatest difference among the cell preparations of all the strains.

Interestingly, the amounts of glucose released by LDL20 were not significantly different between whole cells and their lysates and were among the lowest glucose concentrations quantified for all strains used in this study. The result was especially striking because it had the highest β-gal activities measured by ONPG hydrolysis, possibly indicating a substantial difference in substrate affinities between ONPG and lactose for LDL20 β-gal. In a study comparing the β-gal kinetics of *L. bulgaricus* with different carbohydrates, peak ONPG hydrolysis was reported around 40°C, whereas lactose hydrolysis was highest above 50°C (Nguyen et al., 2012). Peak ONPG hydrolysis for *L. plantarum* was also reported to occur at a slightly lower temperature than peak lactose hydrolysis (Iqbal et al., 2010).

For the HPAEC-PAD profiles of *L. delbrueckii*-treated samples, oligosaccharides besides those eluting with glucose, galactose, or lactose were considered GOS. Overall, more GOS were produced when cell lysates were used, as indicated by the greater number of peaks with higher retention times and greater peak heights (Figure 1). Notably, lysed LB11 and YB1 had the highest HPAEC-PAD total GOS peak heights, and therefore the greatest GOS yields of 47.3% and 53.9%, respectively. Additionally, the whole cells of YB1 also had a high GOS yield of 47.0%. The LB11 lysate even had a significantly greater glucose to galactose ratio compared with its whole cell reaction (*P* < 0.001, 2-way ANOVA; Table 1), indicating increased transgalactosylation of galactose acceptors compared with lactose hydrolysis.

**Figure 1:**
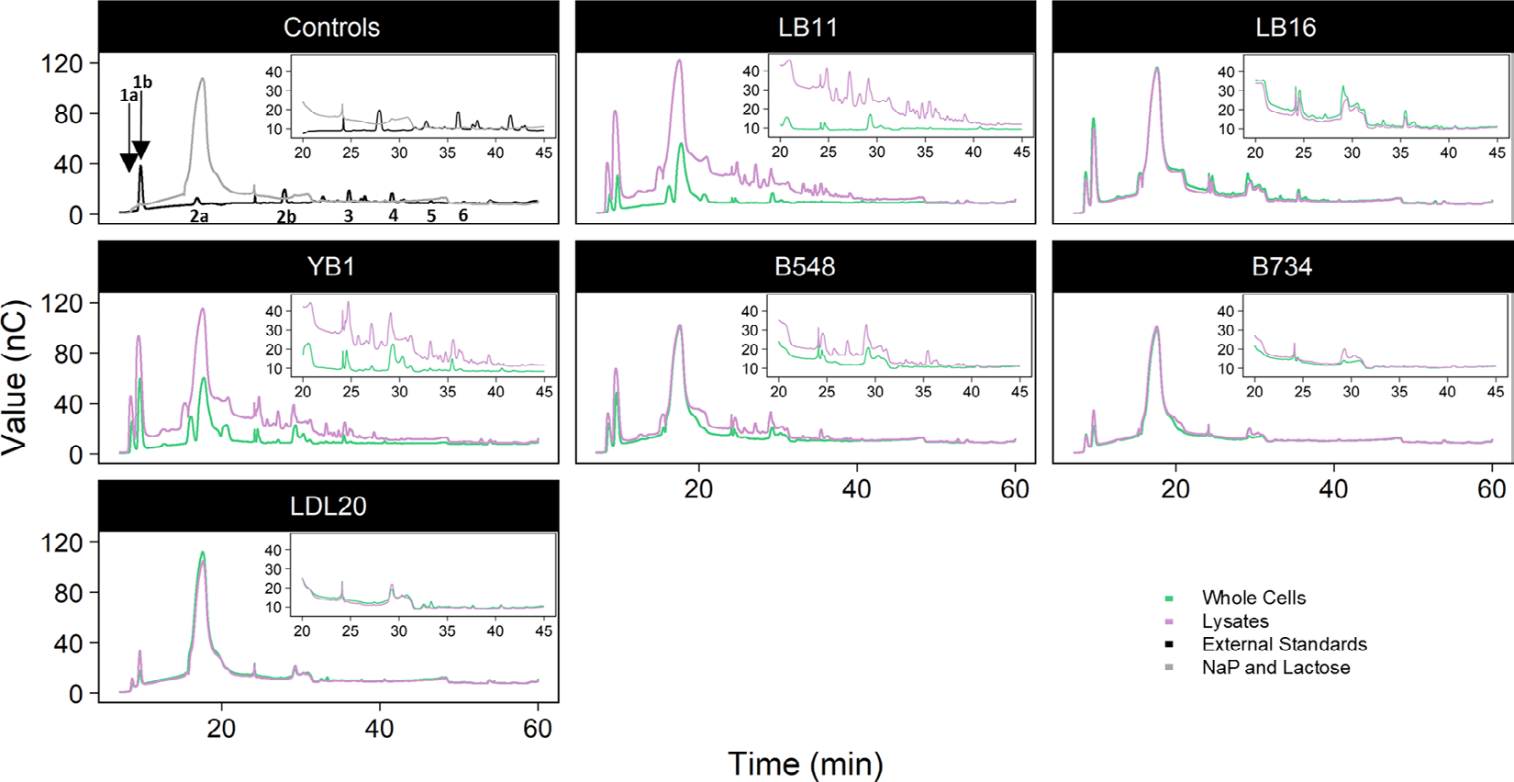
High-performance anion-exchange chromatography with pulsed amperometric detection chromatograms of whole cells and lysates of *Lactobacillus delbrueckii* strains after incubation in 788.8 m*M* lactose in 50 m*M* sodium phosphate (pH 6.5; NaP) for 18 h at 50°C. Numbers indicate external standards included in the run (1a, galactose; 1b, glucose; 2a, lactose; 2b, maltose; 3, malto-triose; 4, malto-tetraose; 5, malto-pentaose; 6, malto-hexaose). Insets were zoomed in between 20 and 45 min. Plots were generated using ggplot2 (Wickham, 2016).

Because LB11 and YB1 produced the greatest GOS yields, they were further tested for GOS production in triplicate for 788.8, 394.4, 131.5, and 0 m*M* lactose in sodium phosphate at 50°C for 18 h. Consistent with the full screen of *L. bulgaricus* strains, the lysates released more glucose than the whole cells (*P* < 0.001, 2-way ANOVA, Table 2), though there were GOS produced by both cell preparations in all concentrations of lactose. The glucose measured from the 0 m*M* lactose reactions averaged 0.1 m*M*. In most cases, lysates also produced more galactose and reduced more lactose than the whole cells.

**Table 2:** Effects of cell preparation at a starting concentration of 7.3 log cfu/mL on β-galactosidase activity in 50 m*M* sodium phosphate (pH 6.5) with differing initial lactose concentrations after 18 h at 50°C^1^

Strain	Initial lactose (m*M*)	Glucose^2^ (m*M*)		Galactose^3^ (m*M*)		Glc:Gal^4^		GOS yield^5^ (%)	
		Whole cells	Lysate	Whole cells	Lysate	Whole cells	Lysate	Whole cells	Lysate
LB11	788.8	30.8 ± 11.7^e^	150.4 ± 11.1^b^	25.1 ± 8.1^cd^	56.0 ± 15.5^b^	1.2 ± 0.2^bc^	2.8 ± 0.6^a^	37.4	47.3
	394.4	64.1 ± 1.5^d^	207.0 ± 8.7^a^	43.8 ± 0.4^bc^	96.1 ± 1.0^a^	1.5 ± 0.0^b^	2.2 ± 0.1^a^	45.1	56.8
	131.5	15.8 ± 3.4^e^	116.0 ± 3.6^c^	20.2 ± 1.6^d^	89.2 ± 0.5^a^	0.8 ± 0.1^c^	1.3 ± 0.0^bc^	41.9	54.9
YB1	788.8	103.9 ± 12.2^y^	190.2 ± 6.2^w^	54.9 ± 10.0^w^	78.7 ± 23.7^w^	1.9 ± 0.1xyz	2.5 ± 0.6^w^	47.0	53.9
	394.4	30.3 ± 0.7^z^	165.5 ± 5.2^x^	21.4 ± 0.4^x^	80.1 ± 3.7^w^	1.4 ± 0.0xyz	2.1 ± 0.0^wx^	43.6	55.1
	131.5	18.5 ± 0.9^z^	103.7 ± 3.9^y^	20.5 ± 0.4^x^	79.9 ± 1.4^w^	0.9 ± 0.0^z^	1.3 ± 0.1^yz^	38.3	53.8

a–e Different letters indicate significant differences among initial lactose and cell preparation of LB11 (*P* < 0.05, 2-way ANOVA, n = 3).

w–z Different letters indicate significant differences among initial lactose and cell preparation of YB1 (*P* < 0.05, 2-way ANOVA, n = 3).

1 Glucose, galactose, and Glc:Gal values are mean ± SD (n = 3).

2 Measured using the Megazyme d-Glucose Assay Kit (GOPOD format; Neogen).

3 Measured using the Megazyme Lactose and d-Galactose (Rapid) Assay Kit (Neogen).

4 Ratio of the mean glucose released to mean galactose released.

5 GOS yield (%) = [GOS peak heights/peak heights (galactose + glucose + lactose + GOS peaks)] × 100; GOS peaks were all peaks excluding galactose, glucose, and lactose.

For YB1, a descending amount of glucose was released with decreasing initial concentrations of lactose. There was only a 1.3% difference in GOS yield between the 131.5 and 788.8 m*M* initial lactose concentrations, indicating that there would be a linear increase in galactose incorporated into GOS dependent on the initial lactose. However, with LB11, there was more free glucose and galactose and a greater GOS yield with an initial lactose concentration of 394.4 m*M* compared with starting with 788.8 m*M*. One previous study using yogurt strains appeared to agree with this finding, where increasing the lactose concentration from 100 to 150 g/L decreased GOS yield from whole cells of *Streptococcus thermophilus* and *L. bulgaricus* yogurt starter cultures (Fischer and Kleinschmidt, 2021). For both strains, release of glucose and galactose strongly correlated with the GOS peak heights (ρ > 0.831, *P* < 0.001, Pearson's), so both free glucose and free galactose can be indicators of GOS production for these strains. There were no noticeable differences in the chromatograms between the 0 m*M* lactose with either strain compared with the initial 0 m*M* lactose solution.

*Lactobacillus delbrueckii* ssp. *bulgaricus* LB11 and YB1 are both able to produce GOS at 50°C at a neutral pH at varying lactose concentrations. LB11 lysate even decreases enzymatic activity above 394.4 m*M* lactose, indicating that greater lactose concentrations do not always substantially increase GOS production. Higher initial lactose concentrations often favor the transgalactosylation activity of β-gal since there should be an abundance of carbohydrates that can serve as acceptors for released galactosyl groups (Iqbal et al., 2022). For example, increasing the initial lactose concentrations for the β-gal reactions of *Lactobacillus acidophilus* between 50 and 450 g/L lactose increased the final GOS by over 100 g/L after 15 h (Carević et al., 2018). For a recombinant β-gal of *L. bulgaricus*, increasing the initial lactose from 80 to 200 g/L resulted in about a 2-fold increase in GOS up to about 70 g/L after 12 h (Arsov et al., 2022).

There are benefits to identifying an enzyme from a strain able to produce GOS for commercial use using a lower initial lactose concentration, like in a dairy product for consumption, especially since lactose should be reduced or eliminated to make product accessible to those with lactose intolerance. The average concentration of bovine milk itself contains about 134 m*M* lactose (Chen et al., 2019), which is close to the 131.5 m*M* used here. The lysates of both LB11 and YB1 produced a GOS yield of 54.9% and 53.8%, respectively, at this lowest initial lactose concentration. Furthermore, the middle concentration of lactose used here at 394.4 m*M* or 13.5% (wt/vol) is within the range of a sugar concentration used in a wide variety of sweetened dairy products. Because the GOS yield of LB11 extracts was optimal at this middle initial lactose concentration, it can be an ideal candidate for food application. However, further studies with different mixtures of carbohydrate sources along with lactose would need to be assessed because food manufacturers do not typically add more lactose to products.

The use of a common starter culture species to produce GOS could benefit the food industry considering the increasing interest in functional ingredients that favor the growth and survival of beneficial bacteria, such as other *Lactobacillaceae* that may benefit from GOS produced by closely related strains. Further studies on LB11 and YB1 should determine the amount of time needed for maximum GOS yields. Further studies should be conducted with the inclusion of other common food ingredients such as milk and sweeteners to determine the feasibility in fermented food products, especially if using live whole cells as the source for β-gal.
